# Overexpression of miR‐483‐5p is confined to metastases and linked to high circulating levels in patients with metastatic pheochromocytoma/paraganglioma

**DOI:** 10.1002/ctm2.260

**Published:** 2020-12-21

**Authors:** Luis Jaime Castro‐Vega, Bruna Calsina, Nelly Burnichon, Tom Drossart, Ángel M Martínez‐Montes, Virginie Verkarre, Laurence Amar, Jérôme Bertherat, Cristina Rodríguez‐Antona, Judith Favier, Mercedes Robledo, Anne‐Paule Gimenez‐Roqueplo

**Affiliations:** ^1^ INSERM, PARCC, Equipe labellisée par la Ligue contre le cancer Paris University Paris France; ^2^ Hereditary Endocrine Cancer Group, Human Cancer Genetics Program Spanish National Cancer Research Centre (CNIO) Madrid Spain; ^3^ Genetics Department, Assistance Publique‐Hôpitaux de Paris Hôpital européen Georges Pompidou Paris France; ^4^ Department of Pathology, Assistance Publique‐Hôpitaux de Paris Hôpital Européen Georges Pompidou Paris France; ^5^ Hypertension unit, Assistance Publique Hôpitaux de Paris Hôpital Européen Georges Pompidou Paris France; ^6^ Paris University, INSERM, Institut Cochin Paris France; ^7^ Rare Adrenal Cancer Network COMETE Paris France; ^8^ Centro de Investigación Biomédica en Red de Enfermedades Raras (CIBERER) Madrid Spain

Dear Editor,

Pheochromocytomas/paragangliomas (PPGL) are tumors of the adrenal medulla and paraganglia. Currently, there is no means to distinguish metastatic from non‐metastatic PPGL based on histopathological criteria. Consequently, patients at risk of progression require a long‐term follow‐up. Here, by conducting complementary analyses of large collections of primary tumors, metastatic tissues, and liquid biopsies, we uncovered that miR‐483‐5p is overexpressed in metastatic tissues compared to primary tumors, whereas the highest levels were detected in the serum of metastatic patients. Further integrative genomic analyses suggest that miR‐483‐5p might be involved in metastasis‐related regulatory networks. These findings pinpoint circulating miR‐483‐5p levels as a promising noninvasive biomarker for the presence of metastasis, which could be useful for guiding patient surveillance.

Although large tumor size, extra‐adrenal location, and germline Succinate Dehydrogenase Complex, Subunit B (*SDHB*) mutations are established risk factors for metastatic PPGL (mPPGL), there is a lack of tumor molecular biomarkers.^1^ In a recent study, we identified a six‐miRNA prognostic signature (miR‐21‐3p, miR‐183‐5p, miR‐96‐5p, miR‐182‐5p, miR‐551b‐3p, and miR‐202‐5p) in primary tumors, and we detected high levels of four of these miRNAs in the circulation of metastatic patients.^2^ To extend the analysis of informative miRNAs, here we evaluated miR‐483‐5p and miR‐210‐3p that have been suggested as biomarkers of mPPGL^3^, ^4^, ^5^, ^6^ and many other tumor types. To this end, the expression of these two miRNAs was interrogated in the largest series of miRNome tumor data (n = 443, n = 7 metastases) published so far (as detailed elsewhere^2^), and validated by Quantitative Reverse Transcription PCR (RT‐qPCR) in an independent series of tumor tissues (n = 107, n = 24 metastases) (Table S1). Circulating levels were assessed in serum from 26 patients and 10 healthy controls using TaqMan assays and droplet digital polymerase chain reaction (PCR). Additional integrative miRNA‐483‐5p‐transcriptome analyses were performed to identify gene sets enriched upon miRNA deregulation and its potential targets.

The expression of miR‐210‐3p was higher in metastatic than in non‐metastatic primary tumors in the published series. However, this result was not reproduced in the validation series (Figure S1) nor in the circulation of metastatic patients, indicating that miR‐210‐3p is not informative for discriminating metastatic from non‐metastatic PPGL. Regarding miR‐483‐5p, we found that its expression did not exhibit significant differences between non‐metastatic and metastatic primary tumors (Figure 1A). However, significant overexpression of miR‐483‐5p was readily detected in metastatic tissues compared to primary tumors in both, published (P = 2.0×10^–3^) and validation series (P = 2.0×10^–5^) (Figure 1A). High expression levels of miR‐483‐5p in metastatic tissues were confirmed in a separated analysis of 17 paired primary‐metastases (*P* = 6.5×10^–4^; Figure 1B). To our knowledge, this is the first time a potential biomarker of mPPGL is evaluated in a large series of metastatic tissues.

**FIGURE 1:**
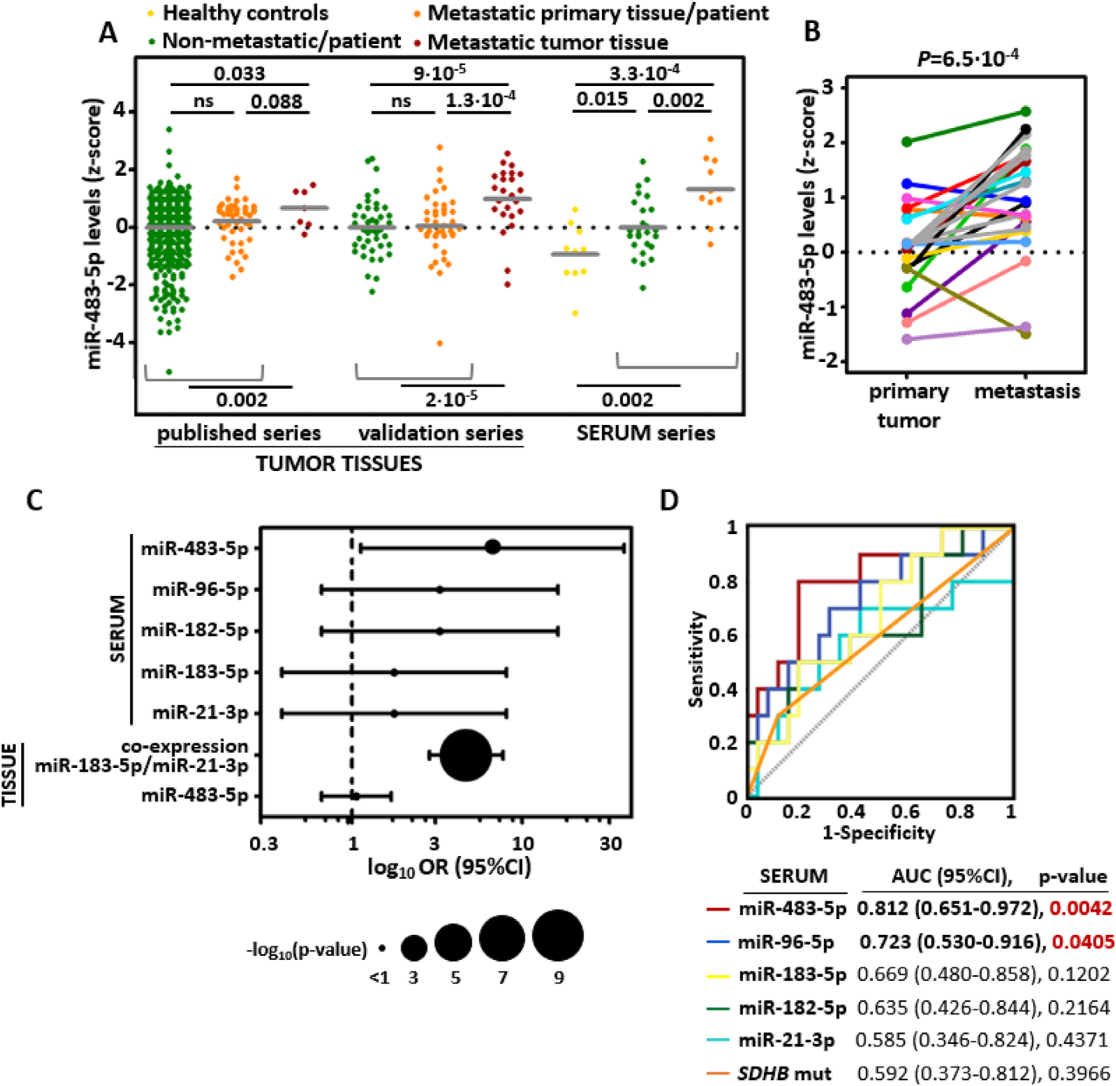
**High levels of miR‐483‐5p are detected in metastases and serum from mPPGL patients**. **A,** Log_2_ normalized expression from the different series is displayed as a transformed z‐score (centered to the mean of non‐metastatic group per each series). Differences in the expression levels in each series were tested by a one‐sided nonparametric Mann‐Whitney test. The mean is shown per each group. **B,** Related to (A), miR‐483‐5p levels in paired primary tumor‐metastatic tissues (n = 17). Each dot represents the level of miRNA in tumor tissue; each color belongs to a different patient. One‐tailed paired *t*‐test was applied to test for differences between paired samples. **C and D,** Comparison of circulating levels of miR‐483‐5p and previously reported miRNAs in mPPGL. Except for miR‐483‐5p, these data were extracted from reported experiments.^2^ **C,** Forest plot showing the odds ratio (OR) obtained from a binary logistic regression applied to test for associations between serum levels of miRNAs and the presence of metastasis in PPGL patients. Also, OR related to risk of metastasis and miRNA expression in tissue is shown for the previously reported classifier (miR‐183‐5p/miR‐21‐3p) (extracted from Calsina et al) and for miR‐483‐5p. The level of each miRNA was expressed as a dichotomous variable using the median level of each miRNA as the cutoff for analyses. Error bars represent the 95% confidence interval (CI), and the diameter of the bubbles is proportional to −log_10_(*P*‐value). For miR‐183‐5p/miR‐21‐3p classifier, the high expression (above median) of both miRNAs at the same time was considered as the cutoff. **D,** Receiver operating characteristic curve analysis showing the accuracy of the different circulating miRNA to discriminate mPPGLs from no‐mPPGL, and *SDHB* status in the serum series

Assessment of circulating miR‐483‐5p in the series of liquid biopsies revealed higher levels in metastatic than in non‐metastatic patients (*P* = .002) (Figure 1A). Accordingly, paired samples from patients with single PPGL for whom blood was collected before surgery, did not show significant correlations of circulating miR‐483‐5p with primary tumor sizes (n = 16; r = 0.142, *P* = .599) nor with its expression in primary tumors (n = 9; r = ‐0.237, P = 0.54). Circulating miR‐483‐5p was the finest marker to indicate the presence of metastases (OR = 6.4, 95% CI = 1.12‐36.44, *P* = .036) and the only one selected after applying a stepwise conditional logistic regression to define the best classifier compared to circulating levels of miRNAs with prognostic value when assessed in primary tumors^2^ (Figure 1C). Further receiver operating characteristic curve (ROC) analysis showed that circulating miR‐483‐5p has the highest accuracy as presence of metastasis discriminator (Area Under the Curve (AUC) = 0.81, 95% CI = 0.651‐0.972, P = 4.0 × 10^–3^). This accuracy was comparable to the reported for miR‐21‐3p/miR‐183‐5p levels in primary tumors (AUC = 0.804, *P* = 4.67 × 10^–18^)^2^ and was largely superior to the *SDHB* status (Figure 1D). Therefore, the miR‐483‐5p appears as the most promising biomarker of mPPGL when assessed in liquid biopsies. Collectively, these data indicate that miR‐483‐5p is likely released at sites of metastatic colonization and may account for the high circulating levels detected in metastatic patients. From the clinical standpoint, circulating miR‐483‐5p could be used to inform when indolent patients become metastatic.

Notably, by scaling miR‐483‐5p expression across 380 metastases from the TCGA consortium, we found that the two metastatic tissues of the PPGL cohort display the highest levels of miR‐483‐5p (Figure S2). According to the human miRNA tissue atlas, the expression of miR‐483‐5p is not tissue specific. Thus, overexpression of this miRNA might play active roles in PPGL cells at the metastatic niche. To explore this possibility, we carried out a comprehensive omics data integration.

Correlation analysis of miR‐483‐5p and gene expression data revealed 152 genes associated with miR‐483‐5p levels (−0.4 > *ρ* > 0.4 and *P* ≤ 1·10^–4^). Functional enrichment analysis suggested miR‐483‐5p as an important regulator of angiogenesis, wound healing, and extracellular matrix organization (Figure 2A), which are tightly related to invasion/metastasis. This finding is in line with the fact that miR‐483‐5p regulates the expression of its host gene IGF2, a stimulator of angiogenesis,^7^ and that *IGF2* expression highly correlates with miR‐483‐5p in PPGL and other cancers^3^ (Figure S3). The involvement of miR‐483‐5p in angiogenesis and extracellular matrix regulation has been demonstrated in models of ischemic disease and osteoarthritis,^8^, ^9^ whereas direct regulation of wound healing by miR‐483‐5p was reported in a model of skin repair.^10^

**FIGURE 2:**
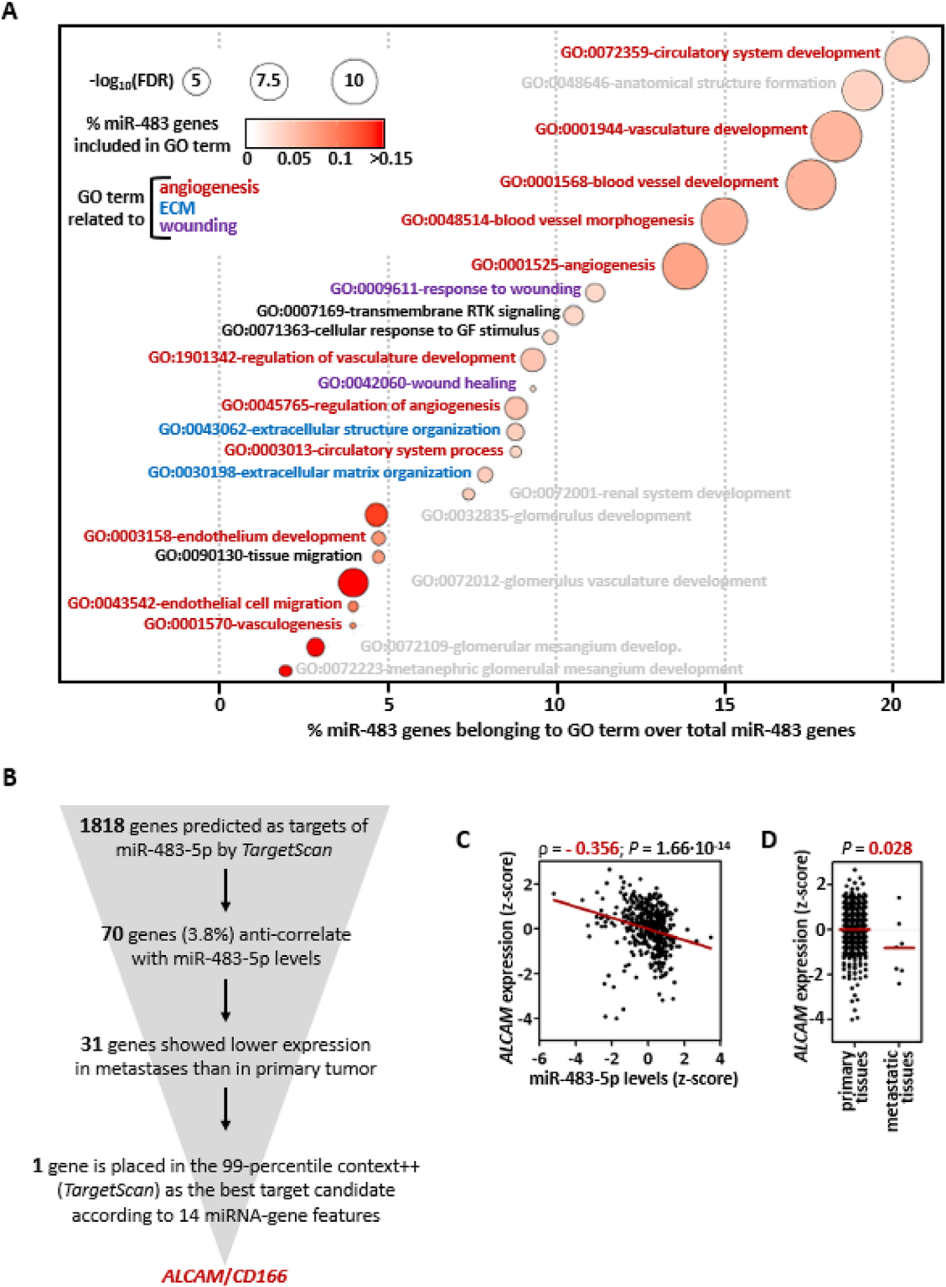
**Functional enrichment and integrative analyses of miR‐483‐5p and transcriptome data. A,** Bubble diagram showing the most significant biological processes obtained after applying STRING (https://string‐db.org/) using the list of genes that correlate significantly with miR‐483‐5p levels. The color of the bubbles indicates the percentage of genes that correlate with miR‐483‐5p levels (here designated as miR‐483 genes) and are included in each specific Gene Ontology (GO) term. The diameter of the bubbles is proportional to the −log_10_(FDR) obtained with STRING tool. The x‐axis shows the percentage of miR‐483 genes that are present in each GO term over the total number of miR‐483 genes. GO terms in red indicate gene sets related to angiogenesis, in blue gene sets related to extracellular matrix (ECM), and in purple gene sets related to wounding. **B,** Workflow and filtering criteria used for analysis of miR‐483‐5p targets, leading to the identification of *ALCAM*. **C,** Scatter plot showing the correlation between miR‐483‐5p and *ALCAM* expression from published series (n = 443). Levels of miR‐483‐5p and *ALCAM* are displayed as a transformed z‐score (centered at the mean of the expression in each series). Spearman's correlation (*ρ*) and *P* values are shown. **D,** *ALCAM* levels in primary tissues and metastases from the published series. Differences in the expression were tested by a one‐sided nonparametric Mann‐Whitney test. The mean is shown per each group

To identify miR‐483‐5p targets that could better explain its potential oncogenic functions, we followed the workflow described in Figure 2B. A single gene, activated leukocyte cell adhesion molecule (*ALCAM)/CD166*, was identified as the best target candidate. *ALCAM* 3’ Untranslated Region (UTR) sequence has an 8 mer‐seed matched site for miR‐483‐5p (Figure S4), and its expression levels significantly anti‐correlate with miR‐483‐5p (n = 443, ρ = −0.356; *P* = 1.66 × 10^–14^; Figure 2C). It has been reported that ALCAM expression decreases with tumor progression,^11^ which entails a poor prognosis. In this regard, we observed a lower expression in metastatic tissues compared to primary tumors in our PPGL series (*P* = .028; Figure 2C), as well as in the TCGA PANCAN cohort (n = 396 metastatic tissues, n = 9712 primary tumors; *P* = 5.8×10^–102^). Since previous reports have demonstrated that miR‐483‐5p directly binds *ALCAM* and regulates its expression in lung adenocarcinoma,^12^ the functional interaction miR‐483‐5p/*ALCAM* may shed light on the understanding of PPGL metastatic niche and warrants further investigation.

In conclusion, while assessment of miR‐183‐5p/miR‐21‐3p in primary tumors improves the stratification of patients at risk of metastasis at the time of diagnosis,^2^ here we suggest circulating miR‐483‐5p, a miRNA potentially related to metastatic colonization, as a promising noninvasive biomarker for the follow‐up of that group of patients. These preliminary results encourage prospective studies aimed at ascertain the clinical relevance of such biomarkers.

## CONFLICT OF INTEREST

The authors declare that there is no conflict of interest that could be perceived as prejudicing the impartiality of the research reported.

## ETHICS APPROVAL AND CONSENT TO PARTICIPATE

This study was conducted ethically in accordance with the Declaration of Helsinki. Tumors from the validation series were collected, thanks to the CNIO Tumor bank with the approval of the institutional review board, and the Bioethics and animal welfare committee of the Carlos III Health Institute. For analysis of liquid biopsies, all patients signed a written consent for the genetic study of the blood and/or tissue samples. This study was approved by the institutional review board (Comité de Protection des Personnes [CPP] Ile de France III, June 2012).

## AUTHOR CONTRIBUTIONS

Conception and supervision: Castro‐Vega and Gimenez‐Roqueplo. Design of the study: Castro‐Vega, Calsina, Robledo, and Gimenez‐Roqueplo. Analysis and interpretation of data: Castro‐Vega, Calsina, Burnichon, Drossart, Martínez‐Montes, Verkarre, Amar, Bertherat, Rodríguez‐Antona, Favier, Robledo, and Gimenez‐Roqueplo. Drafting and revising the manuscript: Castro‐Vega, Calsina, Robledo, and Gimenez‐Roqueplo. Acquisition of the data: Castro‐Vega, Calsina, Burnichon, Drossart, Verkarre, and Amar.

## Supporting information


**Figure S1 Levels of miR‐210‐3p in tumor tissues and liquid biopsies**. Log_2_ normalized expression from the different series is displayed as a transformed z‐score (centered to the mean of non‐metastatic group per each series). The mean is shown per each group. Differences in the expression levels were tested using a one‐sided nonparametric Mann‐Whitney test. For this analysis, only n = 10 metastases were included (those already reported in [2])
**Figure S2 miR‐483‐5p levels in metastases (n = 380) across 12 major cancer types**. TCGA projects with miRNA expression data from metastatic tissues (BRCA, ESCA, HNSC, THCA, SKCM, SARC, PPGL, PRAD, PAAD, CESC, COAD, and BLCA) were included for analysis. Data batch effect‐normalized was retrieved from UCSC Xena browser (https://xenabrowser.net/). Primary tumors origin from those metastases with levels 1.5 x interquartile range above the third quartile (Q3 + 1.5xIQR) are highlighted (SKCM: skin cutaneous melanoma, BRCA: breast invasive carcinoma)
**Figure S3 Scatter plot showing the correlation between miR‐483‐5p and *IGF2* expression in the published series (n = 443)**. Levels of both miR‐483‐5p and *IGF2*, are displayed as a transformed z‐score (centered at the mean of the expression in each series). Data analysis was performed as detailed elsewhere (2). Pearson correlation (*r*) and *P* value are shown.
**Figure S4 TargetScan v7.0‐predicted miR‐483‐5p – *ALCAM* 3’UTR (Untranslated Region) interaction site**. *8mer site type*: perfect Watson‐Crick pairing with the best site efficacy in single miRNA‐gene 3’UTR. *Context ++ score percentile*: score to rank miRNA target predictions considering multiple site‐sequence features
**Table S1 Clinical data of the additional patient samples included in the validation series**. Cases previously described [2] are shown in blue. Additional cases included for this study appear in black. For *primary tumors‐metastases paired* group: Δ = patient with five metastic tissues available (three already included in the previous study, two new samples), * = one of the patients with two metastases available. For *primary tumors from metastatic patients* and *metastases* groups, only tumor tissue from the specified site was available for each patient
Abbreviations: PCC, pheochromocytoma; PGL, paraganglioma; WT, wild type.

## Data Availability

Detailed information of the genomic platforms and cohorts analyzed was reported elsewhere (2).
