# Supplementary material for: Overexpression of miR‐483‐5p is confined to metastases and linked to high circulating levels in patients with metastatic pheochromocytoma/paraganglioma
Source: Clin Transl Med. 2020 Dec 21;10(8):e260. doi: 10.1002/ctm2.260 (PMC7752161; doi:10.1002/ctm2.260)
Supplement: Supplementary file 1 — Figure S1 Levels of miR‐210‐3p in tumor tissues and liquid biopsies. Log2 normalized expression from the different series is displayed as a transformed z‐score (centered to the mean of non‐metastatic group per each series). The mean is shown per each group. Differences in the expression levels were tested using a one‐sided nonparametric Mann‐Whitney test. For this analysis, only n = 10 metastases were included (those already reported in [2]) Figure S2 miR‐483‐5p levels in metastases (n = 380) across 12 major cancer types. TCGA projects with miRNA expression data from metastatic tissues (BRCA, ESCA, HNSC, THCA, SKCM, SARC, PPGL, PRAD, PAAD, CESC, COAD, and BLCA) were included for analysis. Data batch effect‐normalized was retrieved from UCSC Xena browser (https://xenabrowser.net/). Primary tumors origin from those metastases with levels 1.5 x interquartile range above the third quartile (Q3 + 1.5xIQR) are highlighted (SKCM: skin cutaneous melanoma, BRCA: breast invasive carcinoma) Figure S3 Scatter plot showing the correlation between miR‐483‐5p and IGF2 expression in the published series (n = 443). Levels of both miR‐483‐5p and IGF2, are displayed as a transformed z‐score (centered at the mean of the expression in each series). Data analysis was performed as detailed elsewhere (2). Pearson correlation (r) and P value are shown. Figure S4 TargetScan v7.0‐predicted miR‐483‐5p – ALCAM 3’UTR (Untranslated Region) interaction site. 8mer site type: perfect Watson‐Crick pairing with the best site efficacy in single miRNA‐gene 3’UTR. Context ++ score percentile: score to rank miRNA target predictions considering multiple site‐sequence features Table S1 Clinical data of the additional patient samples included in the validation series. Cases previously described [2] are shown in blue. Additional cases included for this study appear in black. For primary tumors‐metastases paired group: Δ = patient with five metastic tissues available (three already included in the previous stu [file CTM2-10-e260-s001.docx]

**Appendix**


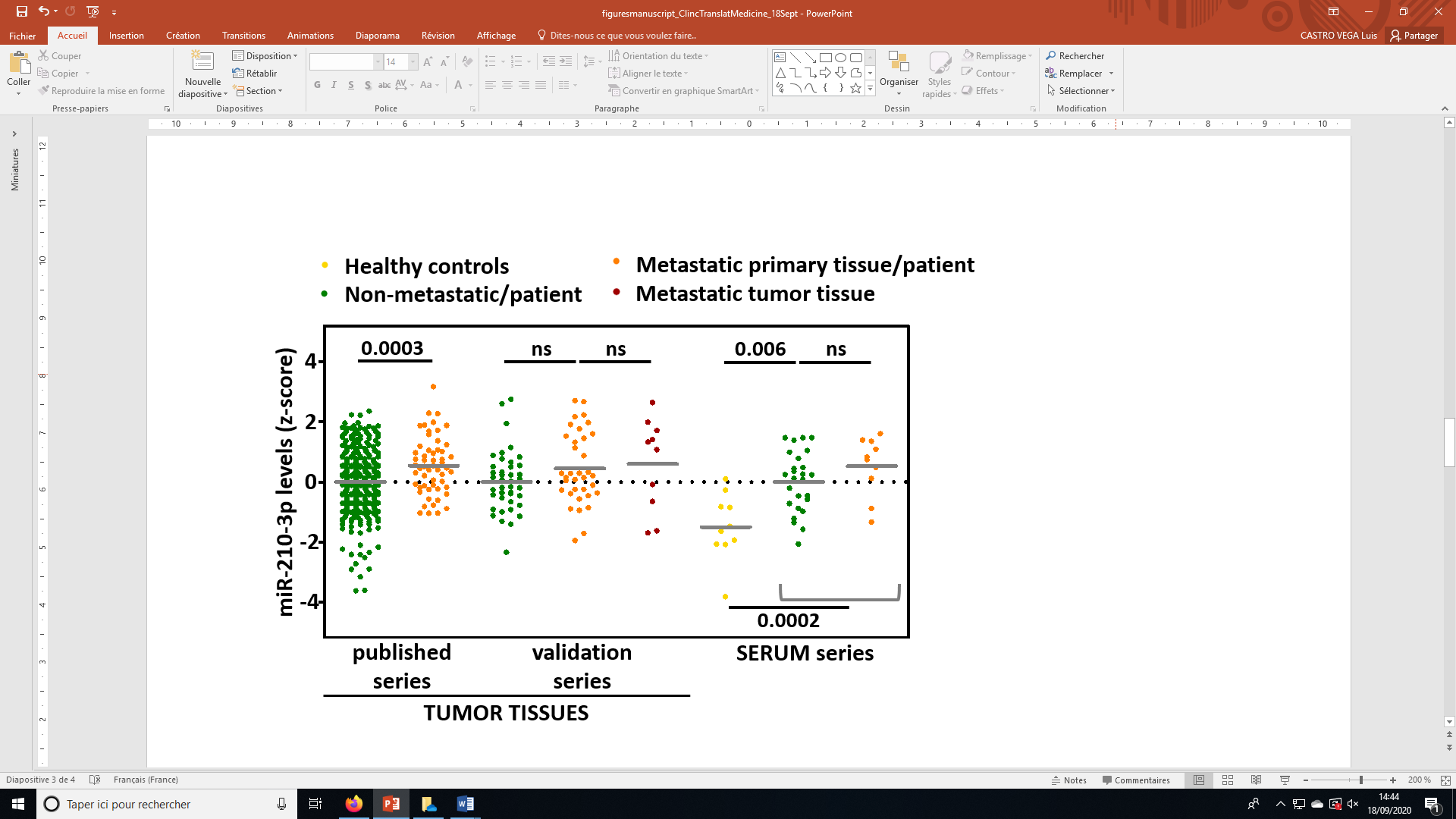


**Fig. S1. Levels of miR-210-3p in tumor tissues and liquid biopsies**. Log_2_ normalized expression from the different series is displayed as a transformed z-score (centered to the mean of non-metastatic group per each series). The mean is shown per each group. Differences in the expression levels were tested using a one-sided nonparametric Mann-Whitney test. For this analysis, only n=10 metastases were included (those already reported in [2]).


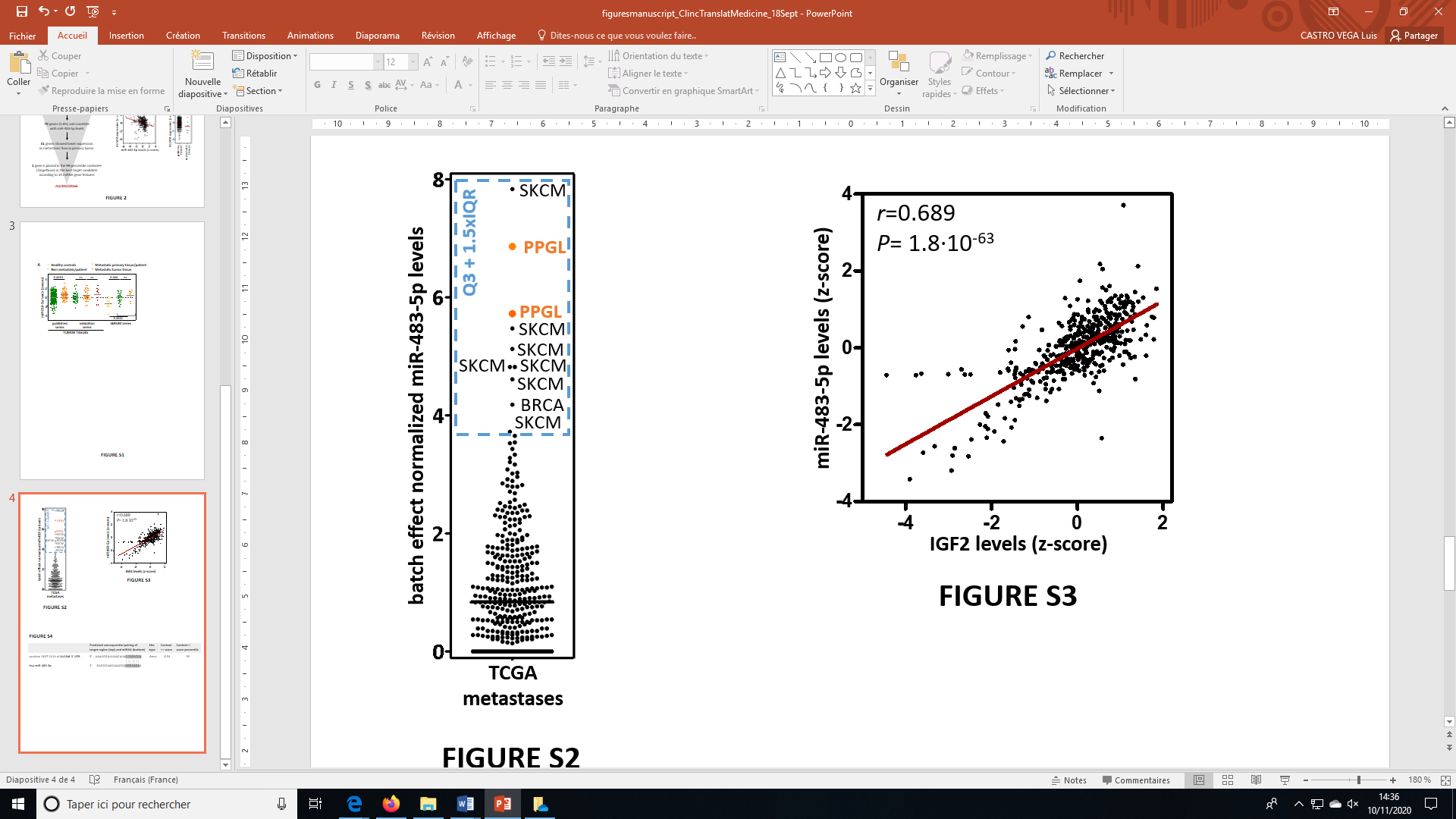


**Fig. S2.** **miR-483-5p levels in metastases (n=380) across 12 major cancer types.** TCGA projects with miRNA expression data from metastatic tissues (BRCA, ESCA, HNSC, THCA, SKCM, SARC, PPGL, PRAD, PAAD, CESC, COAD and BLCA) were included for analysis. Data batch effect-normalized was retrieved from UCSC Xena browser (https://xenabrowser.net/). Primary tumor origin from those metastases with levels 1.5 x interquartile range above the third quartile (Q3 + 1.5xIQR) are highlighted (SKCM: Skin Cutaneous Melanoma, BRCA: Breast Invasive Carcinoma).


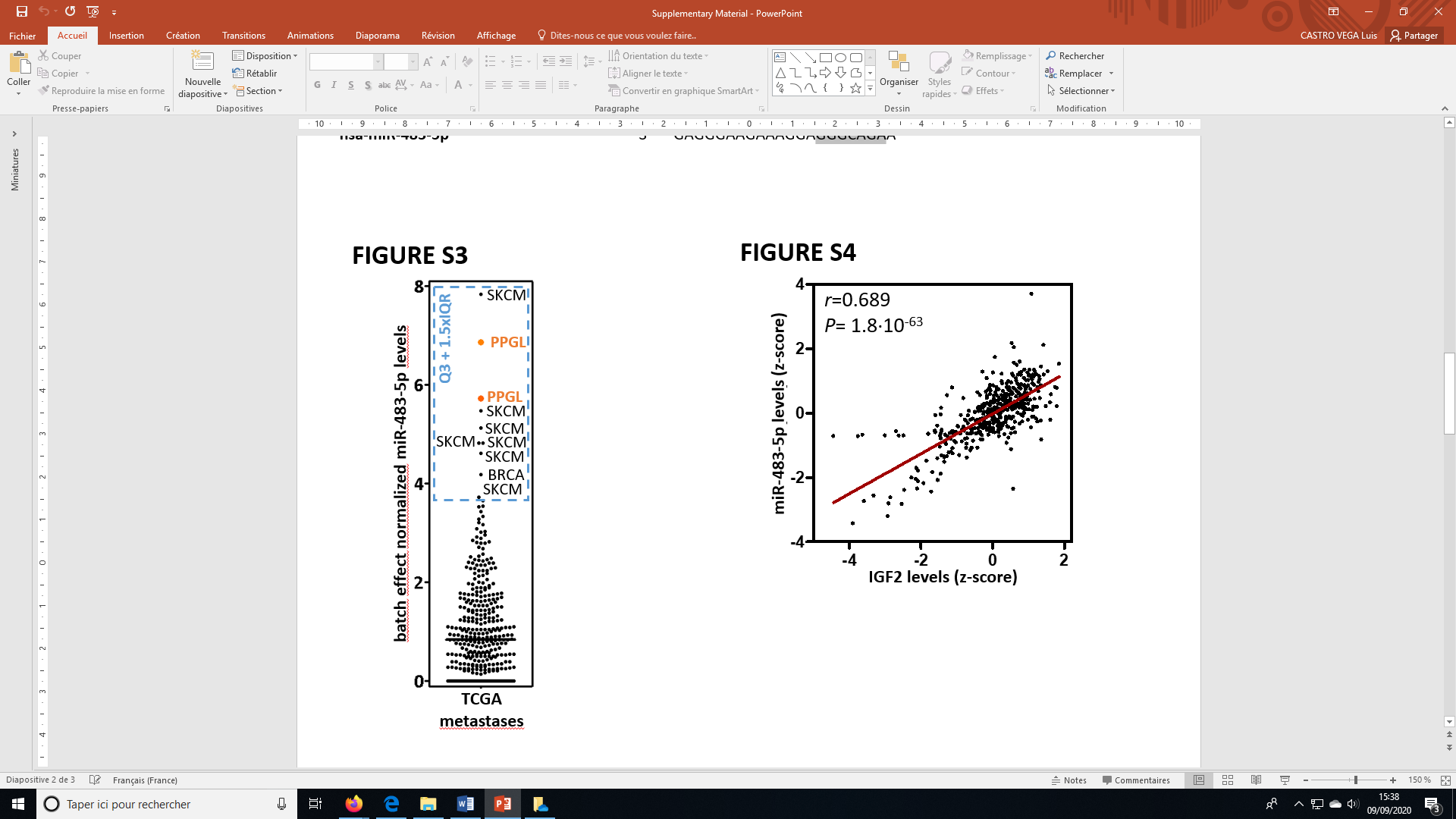


**Fig. S3. Scatter plot showing the correlation between miR-483-5p and *IGF2* expression in the published series (n=443).** Levels of both miR-483-5p and *IGF2*, are displayed as a transformed z-score (centered at the mean of the expression in each series). Data analysis was performed as detailed elsewhere [2]. Pearson correlation (*r*) and *P* value are shown.


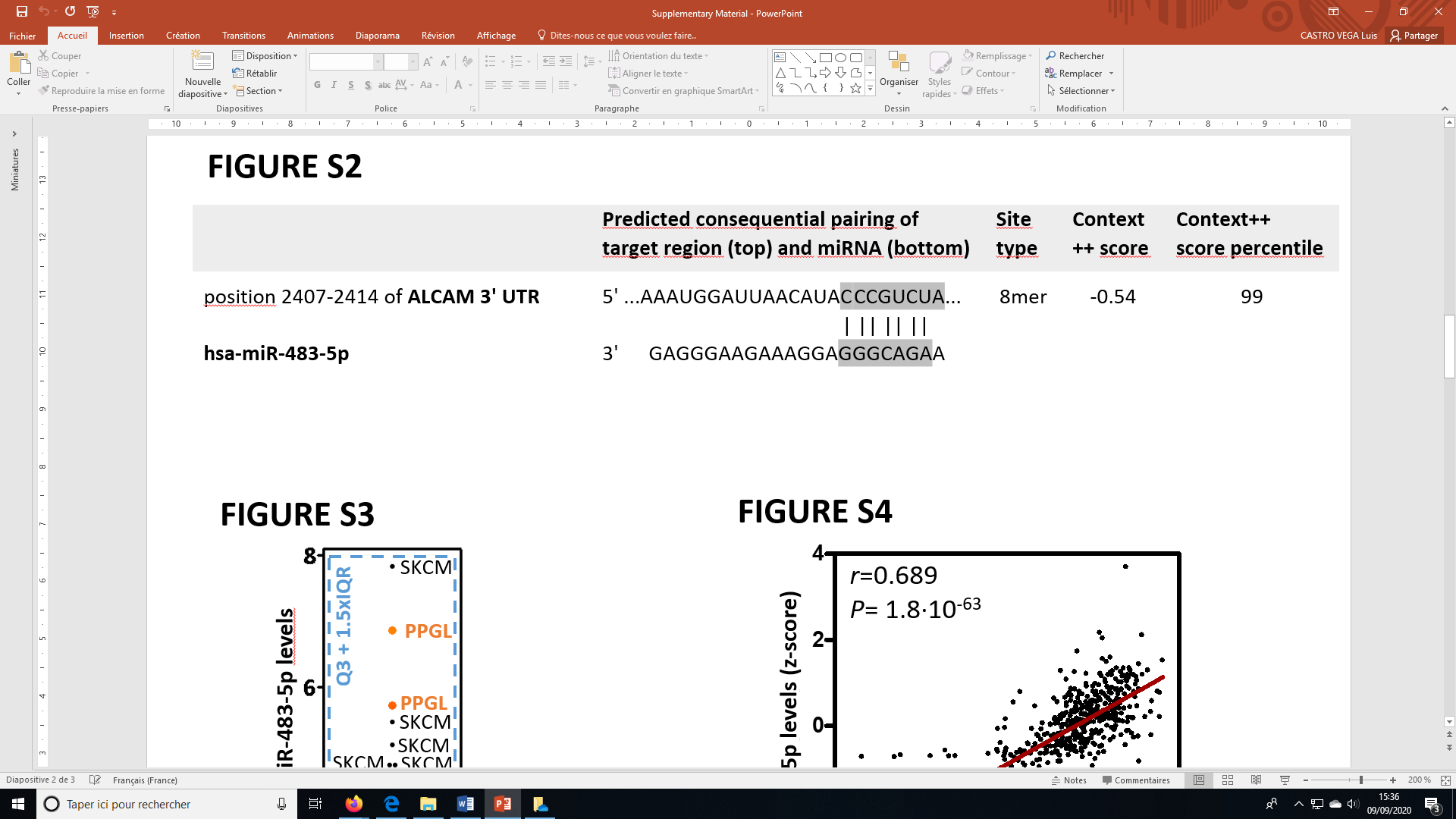


**Fig. S4. TargetScan v7.0-predicted miR-483-5p – *ALCAM* 3’UTR interaction site.** *8mer site type*: perfect Watson-Crick pairing with the best site efficacy in single miRNA-gene 3’UTR. *Context ++ score percentile*: score to rank miRNA target predictions considering multiple site-sequence features.


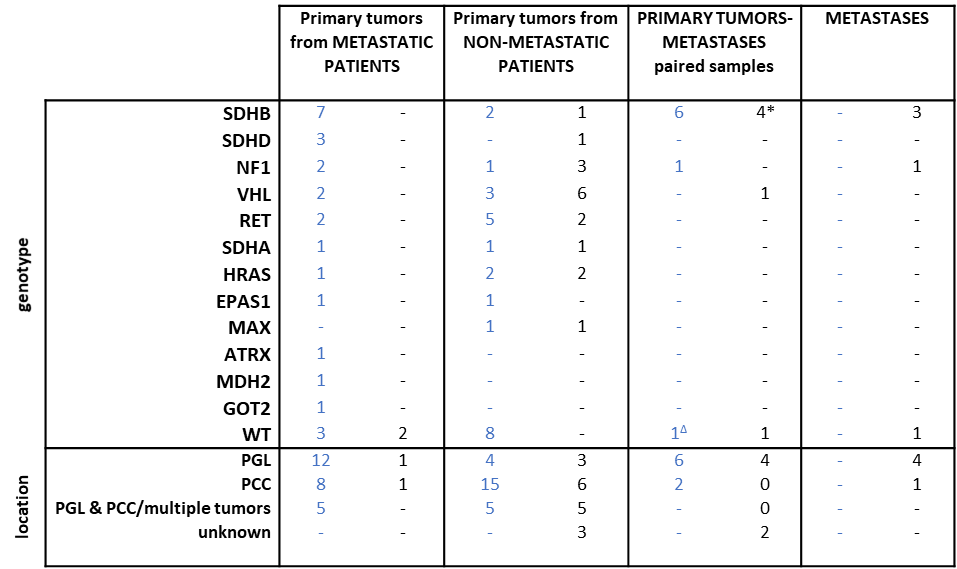


**Table S1. Clinical data of the additional patient samples included in the validation series**

Cases previously described [2] are shown in blue. Additional cases included for this study appear in black. For *primary tumors-metastases paired* group: Δ=patient with 5 metastic tissues available (3 already included in the previous study, 2 new samples), *=one of the patients with 2 metastases available. For *primary tumors from metastatic patients* and *metastases* groups, only tumor tissue from the specified site was available for each patient. WT: wild type; PGL: Paraganglioma; PCC: Pheochromocytoma.
